# Supplementary material for: Calcineurin-mediated regulation of growth-associated protein 43 is essential for neurite and synapse formation and protects against α-synuclein-induced degeneration
Source: Front Aging Neurosci. 2025 Apr 7;17:1566465. doi: 10.3389/fnagi.2025.1566465 (PMC12009912; doi:10.3389/fnagi.2025.1566465)
Supplement: Supplementary file 1 [file Data_Sheet_1.pdf]

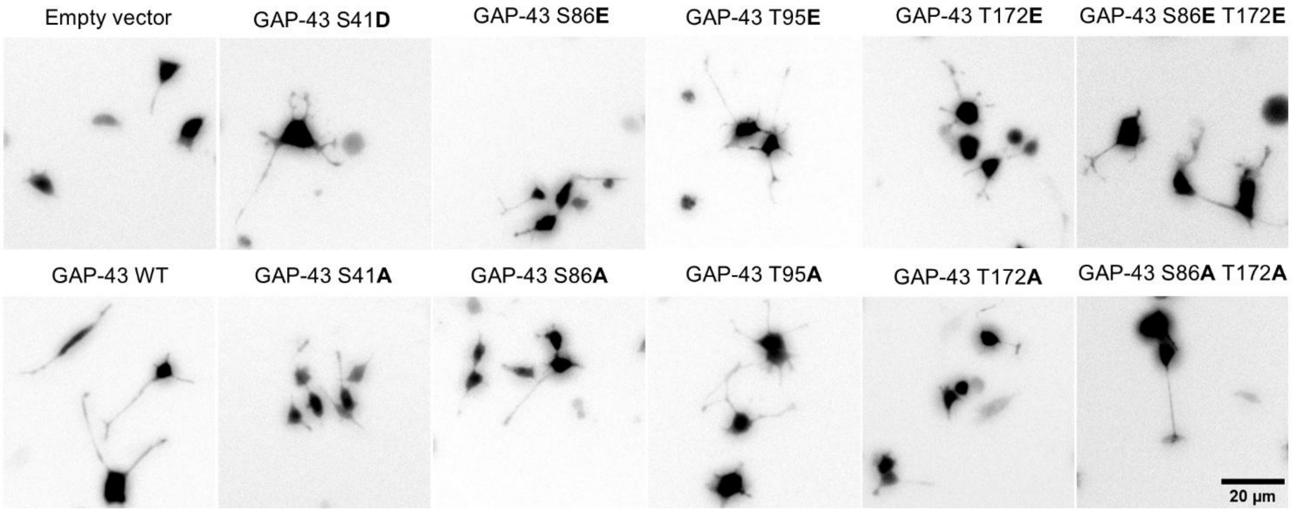

Supplementary Figure 1. Calcineurin-dependent phosphosites S86 and T172 of GAP-43 contribute to neurite branching in PC12 cells. Representative confocal images of PC12 cells co-transfected with GFP for neurite visualization and GAP-43 either WT, phosphomimetic mutants S41D (as a positive control), S86E, T95E, T172E or S86E-T172E double mutant, and phosphoablative mutants S41A, S86A, T95A, T172A or S86A-T172A double mutant. 20×; scale bar is 20µm.
